# High level of depressive symptoms as a barrier to reach an ideal cardiovascular health. The Paris Prospective Study III

**DOI:** 10.1038/srep18951

**Published:** 2016-01-08

**Authors:** B. Gaye, C. Prugger, M. C. Perier, F. Thomas, M. Plichart, C. Guibout, C. Lemogne, B. Pannier, P. Boutouyrie, X. Jouven, J. P. Empana

**Affiliations:** 1 INSERM, UMR-S970, Paris Cardiovascular Research Center, Department of Epidemiology, Paris, France; 2 Université Paris Descartes, Sorbonne Paris Cité, Faculté de Médecine, Paris, France; 3 Preventive and Clinical Investigation Center, Paris, France; 4 APHP, Hospital Broca, Department of Geriatry, Paris, France; 5 APHP, Georges Pompidou European Hospital, Psychiatry Department, Paris, France; 6 INSERM U894, Neuropsychiatry, Paris, France; 7 APHP, Georges Pompidou European Hospital, Pharmacology Departments, Paris, France; 8 APHP, Georges Pompidou European Hospital, Cardiology Department, Paris, France

## Abstract

We hypothesized that depression might represent a barrier to reach an ideal cardiovascular health (CVH) as estimated by the 7-item tool proposed by the American Heart Association. Between 2008 and 2012, 9,417 subjects 50–75 years of age were examined in a large health center and enrolled in the Paris Prospective Study III (PPS3). Participants with 0–2, 3–4 and 5–7 health metrics at the ideal level were categorized as having poor, intermediate and ideal CVH, respectively. Participants with a score ≥7 on the 13-item Questionnaire of Depression 2nd version, Abridged or who were on antidepressants were referred as having high level of depressive symptoms (HLDS). The mean age of the 9417 study participants was 59.57 (SD 6.28) years and 61.16% were males. A total of 9.55% had HLDS. Poor, intermediate and ideal CVH was present in 40.38%, 49.52% and 10.10% of the participants. In multivariate polytomous logistic regression analysis, HLDS was inversely associated with ideal CVH (odds ratio = 0.70; 95% CI: 0.55;0.90). This was driven by an association with the behavioural component of the CVH. Participants with HLDS had a substantial reduced chance of reaching an ideal CVH.

Despite the frank decline of the incidence and mortality associated with cardiovascular diseases (CVD) over these two decades, CVD remain worldwide the first leading cause of mortality^1^. Therefore, continuous efforts on the prevention of CVD must be sustained and the identification of barriers that limit the promotion of cardiovascular health should be a priority. Primary prevention has shown to be effective since the decrease of some traditional risk factors including blood pressure, lipids and smoking (in men) has been suggested to account for almost two-thirds of the decline in CVD death rates^2^. However there remains some concern regarding the trends of other traditional risk factors such as obesity, diabetes and smoking (in women)^3^,^4^. Thus, a complementary approach of the primary prevention of CVD could be to prevent the development of traditional risk factors in first place. This concept of primordial prevention, initially developed by Strasser *et al.*^5^, has been recently re-emphasized by the American Heart Association (AHA)^6^,^7^. In 2010, the AHA has proposed a simplified 7-item tool including 4 health behaviors (smoking, body weight, physical activity, and optimal diet) and 3 biological measures (blood cholesterol, blood glucose, and blood pressure) to promote ideal cardiovascular health (CVH)^6^,^7^. The goal was to increase the prevalence of an ideal CVH in the US population by 20% by 2020 while reducing death from CVD and stroke by 20%. Achieving this goal is of public health importance since recent population-based prospective study cohorts have shown that the risk of CVD was 5-fold lower in subjects with an ideal CVH as compared to those with a poor CVH^8^,^9^,^10^. Unfortunately, the prevalence of an ideal CVH remains low in the US and European populations^8^,^11^,^12^,^13^,^14^,^15^. Therefore, the identification in individuals of potentially modifiable barriers to reach ideal CVH is a major public health priority. Psychological factors may represent one of these barriers. Mild and severe depressive symptoms are very common in the population affecting between 8 and 12% of the general population^16^. Subjects with depressive symptoms may be less likely to reach an ideal CVH for several reasons including (but not restricted to) lack of adherence to health counseling, lack of motivation. Accordingly, the adoption of unhealthy habits including a higher prevalence of smoking and a higher difficulty in smoking cessation, together with a higher prevalence of physical inactivity have been reported^17^,^18^,^19^ and have been suggested to partly explain the much higher rates of CVD in subjects with as compared to subjects without depressive symptoms^20^. Importantly, pharmacological and non-pharmacological strategies such as cognitive behavioural therapies or physical activity have been shown to reduce the severity of depressive symptoms^21^,^22^. Therefore, we hypothesized that high level of depressive symptoms could represent a modifiable barrier to reach an ideal CVH and anticipated that there would be an inverse association between high level of depressive symptoms and ideal CVH.

Using the baseline data of the Paris Prospective Study III including participants aged 50 to 75 years, we quantified the extent to which subjects with high level of depressive symptoms had a lower chance of reaching an ideal CVH.

## Results

### Participant’s general characteristics

Among the 10,157 subjects enrolled in the PPS3, 20 could not be assigned a depressive symptoms status. The number of subjects with missing CVH metrics ranged from 20 (smoking metric) up to 1,330 (diet metric). Nevertheless, a global CVH status could be assigned to 9417 participants. The 740 with missing CVH status were more often women, living alone, current smokers and had less university education (Supplementary Table 1).

The mean age of the 9417 study participants was 59.57 (SD 6.29) years and 61.16% were males. A depressive symptom score ≥7 was observed in 5.50% of the population (3.30% in men and 8.96% in women; p < 0.001) and 5.32% of the population was on antidepressants (3.11% in men and 8.8% in women; p < 0.001). Therefore, a total of 9.55% had HLDS (5.72% in men and 15.58% in women; p < 0.001). Poor, intermediate and ideal CVH was observed in 40.38% (47.14% in men vs. 29.74% in women), 49.52% (45.75% in men and 55% in women) and 10.10% (7.12% in men and 15.25% in women) of the study participants, respectively (all p values for sex comparisons  < 0.001). Only 2.58% of the population had a personal history of CVD.

### Participants’ characteristics by high level of depressive symptoms status

The baseline characteristics of the study participants by HDLS status are reported in Table 1. As expected, participants with HLDS were twice as likely to be women than men. Participants with HLDS were also more likely to be living alone, to have worse self-rated health, and were less educated. They were more likely to have a poor status for all behavioural metrics except diet. Regarding health factors participants with HLDS were more likely to have a poor status for fasting glucose metric only.

### Participants’ characteristics by cardiovascular health status

The baseline characteristics of the study participants by global CVH status are reported in Table 2. With increasing level of CVH, the proportion of women increased while the burden of risk factors decreased significantly; age did not differ accross CVH categories.

### Distribution of cardiovascular health status by high level of depressive symptoms status

**Figure 1:**
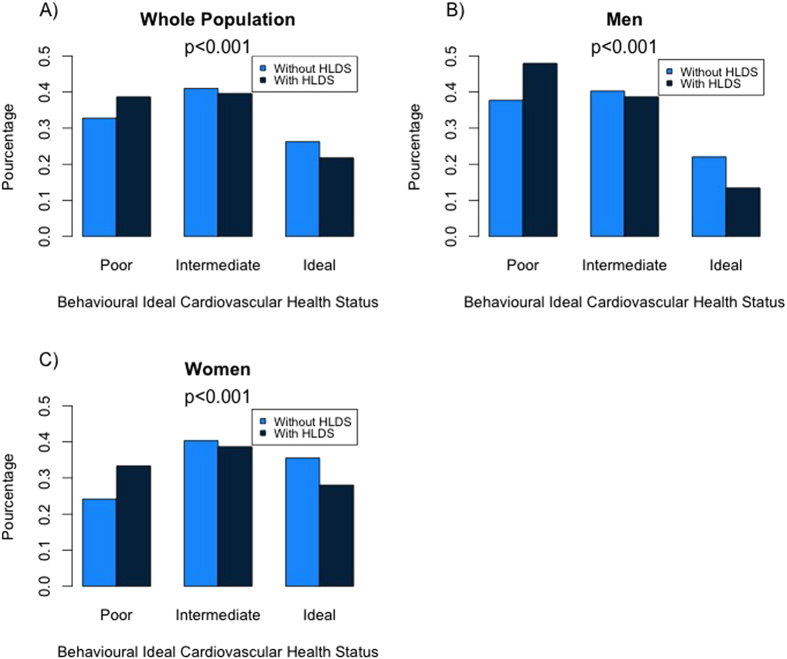
Distribution of the behavioural component of Cardiovascular Health in Paris Prospective Study 3 participants with and without High levels of Depressive Symptoms in the wole population (1A) and by gender (1B,1C).

The distribution of poor, intermediate and ideal behavioural CVH in participants with and without HLDS is reported in Fig. 1. Poor CVH was more frequently observed in subjects with HLDS while ideal CVH was more frequently observed in subjects free of HLDS (Fig. 1A). Sex stratified analysis (Fig. 1B,C) indicates that the prevalence of intermediate and ideal behavioural CVH was systematically higher in women compared to men, whether or not with HLDS. By contrast, there was no significant trend between biological CVH and HLDS even in sex-stratified analyses (not shown). This may explain that trends between *global* CVH and HLDS were not statistically significant (supplementary figure 1).

### Association of HLDS with each metric of CVH

Table 3 listed out the odds ratio for high depressive symptoms for reaching an intermediate and ideal level for each metric of the CVH. After adjustment for age and sex (model 1) subjects with HLDS were significantly less frequently at the intermediate and ideal levels of all behavioural metrics, whereas no associations were reported with any biological metric. Further adjustment for education and living alone (model 2) virtually unchanged these results. The decreased likelihood of being at the intermediate level in the presence of HDLS ranged from 26 (healthy diet) to 33% (physical activity) and that of being at the ideal level ranged from 28 (healthy diet) to 42% (smoking metric).

### Association of HLDS with global, behavioural and biological components of CVH

The separate associations of HLDS with the global, behavioural and biological component of the CVH are reported on Table 4. After adjusting for age and sex (Model 1), participants with HLDS had a 19% (odds ratio = 0.81; 95% confidence interval 0.70–0.94) and a 29% (odds ratio = 0.71; confidence interval 0.56 – 0.90) decreased likelihood of having a *global* CVH at the intermediate and ideal levels. These associations existed with the behavioural component only, with corresponding reduced likelihood of 29% and 45% (p < 0.001). Additional adjustment for education and living alone did not materially change the study results.

Among the 3803 study participants with 2 or less metrics at the ideal levels and categorized as having poor CVH, 1821 (47.88%), 1465 (38.52%) and 71 (1.86%) had respectively 2–3, 4–5 and 6–7 metrics at the intermediate level. Further adjustment for the number of metrics at the intermediate level (model 3, Table 4) yielded even stronger associations between HLDS and the global CVH and its behavioural component. With these additional adjustment, participants with HLDS had a 30% and 44% decreased likelihood of having an intermediate and ideal *global* CVH, and a 36% and 60% decreased likelihood of having an intermediate and ideal behavioural CVH, when compared to those without HLDS.

**Figure 2:**
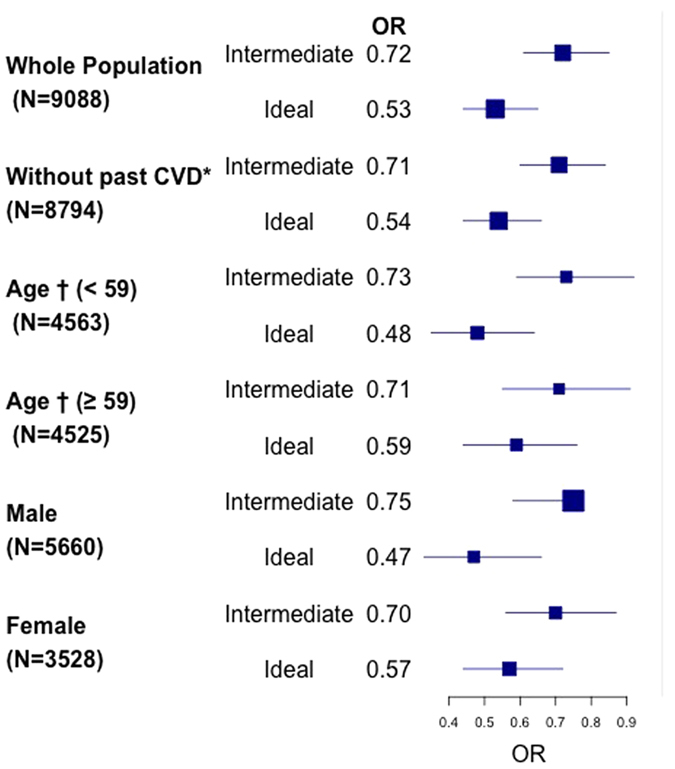
Association between High Level of Depressive Symptoms and the *behavioural* component of Cardiovascular Health: subgroups analysis. *CVD = Cardiovascular Disease; ^†^median.

As shown in Fig. 2, the inverse multivariable association (model 3) between HLDS and the behavioural CVH status was still present after the exclusion of the n = 259 participants with previous CVD, and was consistent across age group and sex. However, the inverse association between HLDS and the ideal (but not the intermediate) behavioural CVH was stronger in women compared to men (p for interaction = 0.07). Similar results were found for the *global* CVH although with lower magnitude (Supplementary figure 2).

### Sensitivity analysis

The results of the sensitivity analyses are detailed in supplementary material. The inverse association between depressive symptoms and *global* and behavioural CVH was confirmed (1) in multivariable linear regression analyses correlating either depressive symptoms score or HLDS status with the global and the behavioural CVH scores; (2) after imputing missing data (depressive symptoms score, confounding variables and CVH metrics) by multiple imputations; (3) after stratification for the country of birth of the participants and of their parents. Furthermore, when exploring the relationship between HLDS and CVH the other way around, we found that compared to those with poor CVH, participants with intermediate and ideal CVH had a significantly lower likelihood of having HLDS in multivariable analysis; this existed for the behavioural component of CVH only. Last, when the n = 93 subjects with a BMI < 18 kg/m^2^ were excluded from the study population, the association between depressive symptoms and the BMI metric, harmoniser CVH was unchanged.

## Discussion

In this cross-sectional analysis of 9417 men and women aged 50 to 75, we explored the hypothesis that high level of depressive symptoms could represent a barrier to reach ideal levels of cardiovascular health. Only 10.10% of the study population reached the required ideal level of cardiovascular health. Further, we observed that subjects with high level of depressive symptoms representing 9.55% of the present study population, had a significant 30% decreased likelihood of being at the ideal level of CVH after accounting for age, sex, living alone and education. This association was observed for the behavioural but not the biological component of the CVH.

Even though our analytic sample is not representative of the general French population aged 50 to 75, that only 10.10% of the study participants had ideal CVH is very consistent with rates published in more representative samples in the US and European populations of comparable age^11^,^12^,^13^,^14^,^15^. After standardization on the age of the European population, the prevalence of ideal CVH was 9.54%. Consistently with other reports, in the present study, women had an ideal cardiovascular health twice more frequently than men. Interestingly, this was observed although they are more frequently depressed than men. The results of our and other studies therefore suggest that additional or specific efforts should be done in men to promote primordial prevention of CVD. Furthermore, despite the variety of instruments used to measure and define the presence of depressive symptoms between studies, the currently reported rate of 9.55% with HLDS is in accordance with what has been previously published in mostly healthy populations of comparable age^16^.

So far, only one study has explored the hypothesis that depression might limit the ability to reach an ideal CVH. In a cross-sectional analysis of the REasonsfor Geopgraphic and Race Differences in Stroke (REGARDS) study conducted in 20,093 US participants aged 45 years and over, Kronish *et al.* reported that the number of health metrics at the ideal level, especially the health behavioural ones, was inversely related to the presence of depressive symptoms^23^. Interestingly, in the REGARDS study, there was an over representation of the stroke belt and of minorities (Afro Americans) and 16% of the study population had experienced previous CHD before study recruitment. Using the PPS3 study, we were therefore able to extend this prior investiagtion in a European context, using a population in whom less than 3% had prevalent CVD. In both the REGARDS and PPS3, the strongest inverse association between depression and the CVH metrics was observed with smoking, which is consistent with the repeatedly reported higher prevalence of smoking and difficulty in smoking cessation in depressed as compared to non-depressed subjects^18^. In PPS3, the second strongest inverse association was observed with the BMI metric whereas in REGARDS, no association was found with this metric. It should be noticed however that in REGARDS one third of the non-depressed were obsese as compared to 9.3% in PPS3.

Another study addressed the hypothesis the other way around and reported that subjects with ideal CVH and specifically ideal behavioural CVH had a significant decreased odds of having depressive symptoms when compared to subjects with poor CVH^24^. Accordingly, our exploratory analyses indicated that subjects with ideal CVH did have a significant and substantial lower likelihood of having HLDS. These results more likely rely on the vascular depression hypothesis^25^, but also indicate that the association between ideal CVH and depression might be bidirectional.

From a methodological point of view, we have seen in those categorized as having a poor CVH a substantial heterogeneity in the distribution of health metrics at the intermediate level. Accounting for this heterogeneity provided even stronger inverse association between HLDS and ideal CVH. This may be an important point for health care providers if one intends to reliably estimate the association between HLDS (and other exposition) and CVH and thus evaluate the benefit on (cardiovascular) health that may be expected by integrating appropriate interventions on depressive symptoms in the population.

### Implications

The growing burden of depressive symptoms in the population and the currently reported 30% decreased likelihood of reaching an ideal CVH in the presence of HLDS suggest that strategies aiming at detecting and treating depressive symptoms in the large segment of the population with poor CVH (40% in the present study) could contribute to enhance the shift from a poor to an ideal CVH. This is a challenging issue but collaborative care targeting simultaneously depressive symptoms and risk factors control might be the most effective and synergistic strategy^26^. Quality of life, compliance to pharmacological treatment and adoption of healthy habits could improve and so CVH^27^, and ultimately cardiovascular disease risk. From our study results, it might be suggested that collaborative care should in particular target simultaneously depressive symptoms and behavioural risk factors control, primarily smoking, obesity and physical inactivity.

### Limitations

We acknowledge the following limitations. Given the cross-sectional design of our analysis, we cannot infer on the temporality regarding the relationship between HLDS and CVH. The age range of our study population between 50 and 75 years limits the generalizability of our study results to other age groups. Furthermore, since the study participants were recruited in the setting of a primary health care center, it is likely that they are in better general health than their counterparts of similar age. This may affect the prevalence of the ideal CVH but not the association between HLDS and CVH. The prevalence of both HLDS and CVH may differ by migrant status and ethnicity^23^ but such informations cannot be obtained in France by law, even for research purposes. However sensitivity analyses indicates that the association between HLDS and ideal CVH was consistent across the country of birth of the participants and of their parents. Furthermore, our analysis relied on HLDS but not on a clinical diagnosis of depression. Leisure physical activity was based on two simple questions and we favored specificity to define this item. It is highly likely that we have underestimated the true level of ideal cardiovascular health for this metric. Last, we lacked data on dietary fiber intake contributing to have incomplete information on the dietary metric of the CVH.

To conclude, in this large French study population, participants with high level of depressive symptoms had a substantial reduced likelihood of reaching intermediate and ideal CVH, especially its behavioural component. These results obtained in a European context extend those recently reported in a US environment (REGARDS study). Therefore, recognition and appropriate interventions on depressive symptoms could help to enhance the shift from poor to ideal CVH in the population.

## Methods

### Study Population

The design and main objectives of the PPS3 have been previously published^28^. It is an ongoing prospective observational cohort studying the contribution of rhythmic parameters, carotid stiffness metrics, and blood biomarkers to the onset of main phenotypes of cardiovascular disease in initially mostly healthy subjects. Our study was registered in the World Health Organization international clinical trial registry platform (NCT00741728) in 25/08/2008. The study protocol was approved by the Ethics Committee of the Cochin Hospital (Paris). The methods were carried out in accordance with the approved guidelines. Between June, 2008 and May, 2012, 10,157 men and women aged 50–75 years were recruited in a large preventive medical center, the Centre d’Investigations Préventives et Cliniques (IPC), in Paris (France) after signing an informed consent form. The IPC is a preventive medical center that is subsidized by the French National Insurance System for Salaried Workers (Sécurité Sociale-CNAMTS), which offers to all working and retired employees and their families a free medical examination every five years. It is one of the largest medical centers of this kind in France, having carried out approximately 20,000–25,000 health examinations per year since 1970 for people living in the Paris area. The standard health check-up includes a complete clinical examination including measurement of height, weight and blood pressure, coupled with standard biological tests after an overnight fasting. A self-administered questionnaire provides information related to professional activity, lifestyle (tobacco and alcohol consumption, physical activity, diet), personal and family medical history, current health status, and medications consumption^29^.

### Height and weight

Anthropometric factors were measured in subjects with light clothing and no shoes by nurses during physical examination. Height was measured to the nearest 0.5 cm with a wall-mounted stadiometer while body weight was measured using calibrated scales. Body mass index (BMI) was quantified as body weight divided by the square of height (kg/m^2^). Poor, intermediate and ideal BMI was defined by values >30 kg/m^2^, between 25 and 29.9 kg/m^2^ and between 18 and 25 kg/m^2^ respectively.

### Smoking habits

In the general self-administrated questionnaire, participants reported their current smoking status (never smoker/ex smoker/current smoker) and either the time since smoking or the time since smoking cessation (for ex smokers). Poor, intermediate and ideal smoking status corresponded to current smokers, ex smokers that have stopped since less than 12 months, and never smokers or ex smokers that have stopped since 12 months or more respectively.

### Blood pressure

Supine blood pressure was measured during carotid echotracking using an oscillometric method (OMRON 705C). Poor, intermediate and ideal blood pressure corresponded to values > 120/80 mm-Hg, values < 120/80 mm-Hg on medications, and untreated 120/80 mm-Hg respectively.

### Blood total cholesterol and glucose

Standard biological tests were performed after an overnight fasting. In particular, total cholesterol and glycaemia were measured using standardized enzymatic methods (automat HITACHI 917, Hitachi, Tokyo, Japan). Poor, intermediate and ideal total cholesterol corresponded to values > 5.18 mmol/L, values < 5.18 mmol/L on medications and untreated values < 5.18 mmol/L respectively. Corresponding thresholds for poor, intermediate and ideal fasting glucose were >5.55 mmol/L, <5.55 mmol/L on medications and untreated values < 5.55 mmol/L respectively.

### Physical activity

In the general self-administrated questionnaire, participants were asked to report (a) whether or not they were practicing a regular physical activity corresponding to more than one hour of walking every day and (b) the frequency with which they were practicing sports (never, twice or less a week, three times or more a week). Participants who were walking at least one hour every day or who were practicing sports 3 times or more per week were categorized has having an ideal physical activity, whereas participants who where practicing sports twice or less a week only were categorized as having an intermediate level of physical activity, and the others as having a poor level of physical activity.

### Habitual food intakes

Habitual food intakes were evaluated using an adapted version of a short self-reported food frequency questionnaire, the NAQA (18 item), designed for use in epidemiological studies and that has been validated against a dietetic interview which used the historic diet method^30^. The questionnaire is detailed in supplementary materials. Information on dietary fiber intake was not available, so that the healthy diet metric was based on the fruits-vegetables, fish, sugar and sodium intakes. Therefore, participants consuming (1) vegetables and fresh fruits more than four times a week, and (2) fish twice or more a week and (3) salt less than 1500mg per day and (4) who never drink any sugar-sweetened beverages were categorized as ideal for this metric. Those combining 2 or 3 of these criteria were categorized as intermediate, and the remaining as poor.

### Cardiovascular Health

As defined above, each metric of the CVH was categorized in three levels as poor, intermediate and ideal. Participants who had 0–2, 3–4 and 5–7 health metrics at the ideal level were defined as having poor, intermediate and ideal ***global*** CVH, respectively^6^. Furthermore, those with 0–1, 2 and 3–4 health behavior metrics at the ideal level were defined as having poor, intermediate and ideal behavioural CVH. Those with 0–1, 2 and 3 health factors at the ideal level were defined as having poor, intermediate and ideal ***biological***CVH^6^. Moreover, participants with prevalent CVD or who were under medications and with 5 or more metrics at the ideal level were categorized as having an intermediate ***global*** CVH; otherwise they were categorized as having a poor global CVH.

The score of CVH was also calculated as follows: each of the seven metrics was assigned a weight (1, 2, or 3 for poor, intermediate, or ideal level, respectively). The sum of these weights could give a score ranging from 7 (all metrics at the poor level) to 21 (all metrics at the ideal level) for global CVH. Similarly, the score for the behavioural CVH could range from 4 (all metrics at the poor level) to 16 (all metrics at the ideal level) and that of the ***biological*** CVH from 3 (all metrics at the poor level) to 9 (all metrics at the ideal level).

### Depressive Symptoms and antidepressants

Depressive symptoms were measured with the 13-item Questionnaire of Depression 2nd version, Abridged (QD2A)^31^. Built on well-established questionnaires such as the Beck Depression Inventory^32^ and the Zung self-rating depression scale^33^, the QD2A has been specifically designed for depression screening in community studies. Participants had to give a yes/no answer to each of the 13- item regarding their current emotional state (e.g. “I am disappointed and disgusted with myself”, “I am sad these days”, “I feel hopeless about the future”). The number of yes answers is summed to provide a total score with high internal consistency (α = 0.91). A total score ≥7 indicates a high probability for major depression (sensitivity: 81%, specificity: 96%)^31^. Study participants were also asked to self- report whether or not they had already suffered from a previous episode of depression. In addition, the IPC questionnaire asked whether or not the participants were currently taking medications for a series of chronic conditions including depression. To reduce under reporting, participants were asked to come at the IPC with either their most recent medical prescriptions and/or with their medical package. Medications were checked by a medical doctor from the IPC. Medications were coded using the World Health Organization (WHO) Anatomical Therapeutic Chemical (ATC) classification. Therefore, study participants with a QD2A ≥7 or who were on antidepressants were referred as having high level of depressive symptoms (HLDS).

### Statistical Analysis

The baseline characteristics of study participants were compared according to their HLDS status and according to their CVH status, using Pearson chi-square test, t-test or one-way analysis of variance where appropriate. Polytomous logistic regression was used to quantify the association of HLDS (main exposure variable) with intermediate and ideal CVH (dependent variable), using poor CVH as the reference category. Participants with missing health metrics were excluded for the analysis of each metric. However, for the analysis of global CVH and of its behavioural and biological components, they were not systematically excluded when the available information on the other metrics was sufficient to assign a global CVH status. For example a participant with 5 ideal health metrics and 2 missing ones could already be categorized as having an ideal global CVH. By constrast, if a participant had 2 health metrics at the ideal level but missing data for the other 5, a global CVH could not be assigned accurately and the participant excluded from the analysis. Regression models were adjusted for age and sex (Model 1) and then for living alone status and educational level (Model 2). To account for the heterogeneous distribution of the metrics at the intermediate levels among participants categorized as having a poor CVH, the analysis was further adjusted for the number of metrics at the intermediate level (Model 3). We tested whether the association between HLDS and ideal CVH differed by age group (according to the median) and sex, using a p value of less than 0.10 to signify relevant interactions. We also re-run the analysis after excluding participants with a personal history of CVD to evaluate residual confounding^34^. Sensitivity analyses were conducted to assess the robustness of our findings and are detailed in supplementary materials. First we considered the QD2A scale and the CVH score as ordinal variables and run linear regression analyses adjusted for age, sex, living alone status and educational level. Second, we re-run our main analysis after imputing all missing data by multiple imputations using chained equation^35^. Third, to address whether the investigated relationship might differ according to the migrant status or the ethnic group of the participants, stratified analyses were conducted. The country of birth of the participants and of their parents, were used as proxies for the migrant status and the ethnic group respectively. Four, although not directly our study hypothesis, we explored the relationship the other way around, i.e. whether ideal CVH (main exposure variable) was associated with lower odds of having HLDS (outcome variable), to evaluate the possibility of reverse causality. Five, given that a BMI < 18 kg/m^2^ might reflect an underlying severe condition, we have repeated the association of BMI metric, behavioural cardiovascular health and global cardiovascular health after excluding the n = 93 subjects with a BMI < 18 kg/m^2^. All statistical analyses were two-tailed and used a p value of less than 0.05 to signify statistically significant associations except for interaction analysis (p < 0.10). Statistical analyses were performed using R 3.1.0.

## Additional Information

**How to cite this article**: Gaye, B. *et al.* High level of depressive symptoms as a barrier to reach an ideal cardiovascular health.The Paris Prospective Study III. *Sci. Rep.* **6**, 18951; doi: 10.1038/srep18951 (2016).

## Supplementary Material

Supplementary Information

## Figures and Tables

**Table 1:** Characteristics of Paris Prospective Study III study participants with and without High Level of Depressive Symptoms (HLDS)*.

Variables	Without High Level of Depressive Symptoms* N = 9163	High Level of Depressive Symptoms* N = 974	p-value^†^
Age	59.61 (±6.30)	59.60 (±6.30)	*0.90*
Men	5833 (63.66%)	357 (36.65%)	*0.001*
University education	6268 (69.00%)	597 (62.45%)	*0.001*
Living Alone	2245 (24.60%)	426 (43.92%)	*0.001*
Self Rated Health	7.52 (±1.66)	6.32 (±1.78)	*0.001*
Smoking			*0.001*
Poor	1305 (14.27%)	214 (21.99%)	
Intermediate	354 (3.87%)	35 (3.60%)	
Ideal	7485 (81.86%)	724 (74.41%)	
Body Mass Index			*0.001*
Poor	851 (9.31%)	125 (12.86%)	
Intermediate	3654 (39.98%)	337 (34.67%)	
Ideal	4635 (50.71%)	510 (52.47%)	
Physical Activity			*0.001*
Poor	2837 (31.22%)	378 (39.13%)	
Intermediate	1731 (19.05%)	165 (17.08%)	
Ideal	4520 (49.74%)	423 (43.79%)	
Healthy Diet			*0.25*
Poor	3461 (43.29%)	379 (45.55%)	
Intermediate	4060 (50.78%)	398 (47.84%)	
Ideal	474 (5.93%)	55 (6.61%)	
Fasting Total Cholesterol			*0.18*
Poor	2669 (29.27%)	304 (31.31%)	
Intermediate	4467 (48.99%)	478 (49.23%)	
Ideal	1983 (21.75%)	189 (19.46%)	
Blood Pressure			*0.30*
Poor	2553 (28.12%)	253 (26.27%)	
Intermediate	4597 (50.64%)	488 (50.67%)	
Ideal	1928 (21.24%)	222 (23.05%)	
Fasting serum glucose			*0.001*
Poor	277 (3.04%)	31 (3.19%)	
Intermediate	4072 (44.63%)	367 (37.80%)	
Ideal	4774 (52.33%)	573 (59.01%)	

Data are reported as n (%) and mean (± standard deviation) for categorical and continuous variables respectively.

*High Level of Depressive Symptoms defined as Questionnaire of Depression 2nd version, Abridged depressive symptoms score ≥7 or being under antidepressant.

^†^p-value for a chi-squared test or t-test where appropriate.

**Table 2:** Characteristics of Paris Prospective Study III study participants by cardiovascular health status.

Variables	Poor (40.38%)	Intermediate (49.52%)	Ideal (10.10%)	p-value*
Age	59.5 (±6.13)	59.7 (±6.40)	59.27(±6.32)	0.14
Men	2715 (71.4%)	2641 (56.6%)	403 (42.3%)	<0.001
University education	2514 (66.7%)	3257 (72.8%)	688 (72.8%)	0.01
Self Rated Health	7.24 (±1.74)	7.51 (±1.69)	7.64 (±1.76)	<0.001
Living Alone	896 (23.6%)	1260 (27.1%)	289 (30.5%)	<0.001
Body Mass Index	27.3 (±3.50)	24.1 (±3.19)	22.22 (±2.35)	<0.001
Smoking _*Ideal*_	2638 (69.40%)	4084 (87.6%)	917 (96.4%)	<0.001
Physical Activity_*Ideal*_	1039 (27.5%)	2725 (58.7%)	808 (85.01%)	<0.001
Fruits and Vegetables_*Ideal*_	1064 (29.5%)	1817 (41.9%)	491 (57.2%)	<0.001
Fish_*Ideal*_	1400 (38.5%)	1903 (43.6%)	476 (55%)	<0.001
Sugar_*Ideal*_	2000 (55.3%)	2534 (58.3%)	582 (67.4%)	<0.001
Sodium_*Ideal*_	1100 (30.8%)	1552 (36.4%)	459 (53.8%)	<0.001
Fasting Total Cholesterol mg/dl	228.3 (±35.85)	219 (±35.5)	204 (±35.22)	<0.001
Systolic Blood Pressure mmHg	136 (±15)	130 (±16)	118 (±13.8)	<0.001
Fasting Serum Glucose mg/dl	106 (±16.3)	97.6 (±12)	92.8 (±6.16)	<0.001
Personal history of CVD^†^	122 (3.22%)	119 (2.58%)	0 (0%)	<0.001
Treatment for blood pressure	731 (19.2%)	534 (11.5%)	34 (3.58%)	<0.001
Treatment for lipids	690 (18.3%)	475 (10.2%)	40 (4.21%)	<0.001

Data are reported as n (%) and mean (+/− standard deviation) for categorical and continuous variables respectively.

Poor, intermediate and ideal CVH are defined as having 0–2, 3–4 and 5–7 health metrics at the ideal level, respectively.

*p-value for a chi-squared test for trend or ANOVA were appropriate.

^†^CVD: Cardiovascular disease.

**Table 3:** Odds ratios for high level of depressive symptoms* to be at intermediate and ideal level of each metric of cardiovascular health.

Outcomes	OR	Unadjusted [95% CI]	*p-value*^†^	OR	Model 1 [95% CI]	*p-value*^†^	OR	Model 2 [95% CI]	*p-value*^†^
Smoking									
Poor	1			1			1		
Intermediate	0.60	[0.49–0.88]	0.008	0.63	[0.42–0.91]	0.016	0.69	[0.57–0.84]	<0.001
Ideal	0.59	[0.49–0.70]	<0.001	0.53	[0.45–0.63]	<0.001	0.58	[0.49–0.69]	<0.001
Body Mass Index									
Poor	1			1			1		
Intermediate	0.63	[0.51–0.78]	0.001	0.70	[0.55–0.87]	<0.001	0.72	[0.57–0.91]	0.005
Ideal	0.75	[0.61–0.93]	0.007	0.63	[0.51–0.78]	<0.001	0.65	[0.52–0.81]	<0.001
Physical Activity									
Poor	1			1			1		
Intermediate	0.71	[0.60–0.87]	0.001	0.67	[0.55–0.82]	<0.001	0.67	[0.55–0.82]	<0.001
Ideal	0.70	[0.61–0.81]	0.001	0.71	[0.61–0.82]	<0.001	0.71	[0.61–0.82]	<0.001
Healthy Diet									
Poor	1			1			1		
Intermediate	0.89	[0.77–1.04]	0.14	0.72	[0.62–0.84]	<0.001	0.74	[0.63–0.86]	<0.001
Ideal	1.05	[0.78–1.43]	0.71	0.72	[0.53–0.98]	0.03	0.72	[0.53–0.98]	0.04
Fasting Total Cholesterol									
Poor	1			1			1		
Intermediate	0.93	[0.81–1.09]	0.41	1.04	[0.86–1.27]	0.63	1.07	[0.91–1.24]	0.42
Ideal	0.83	[0.69–1.01]	0.06	1.06	[0.91–1.24]	0.43	1.02	[0.83–1.24]	0.82
Blood Pressure									
Poor	1			1			1		
Intermediate	1.07	[0.91–1.25]	0.39	1.03	[0.87–1.21]	0.70	1.08	[0.91–1.27]	0.35
Ideal	1.16	[0.96–1.40]	0.12	0.89	[0.74–1.09]	0.81	0.95	[0.77–1.16]	0.63
Fasting Serum Glucose									
Poor	1			1			1		
Intermediate	0.80	[0.55–1.18]	0.27	0.69	[0.47–1.02]	0.06	0.75	[0.50–1.13]	0.17
Ideal	1.07	[0.73–1.57]	0.71	0.73	[0.49–1.07]	0.11	0.78	[0.52–1.17]	0.24

Model 1: Adjusted for **Age** and **Gender**; Model 2 : Model 1 plus **education** and **living alone**.

*High Level of Depressive Symptoms defined as Questionnaire of Depression 2nd version, Abridged depressive symptoms score ≥7 or being under antidepressant.

^†^p-value for polytomous logistic regression.

Odds ratio and 95% Confidence Interval limits were obtained by polytomous logistic regression.

**Table 4:** Odds ratios of high level of depressive symptoms* for intermediate and ideal global, behavioural and biological Cardiovascular Health.

Outcomes	OR	Unadjusted [95% CI]	*p-value*^†^	OR	Model 1 [95% CI]	*p-value*^†^	OR	Model 2 [95% CI]	*p-value*^†^	OR	Model 3 [95% CI]	*p-value*^†^
All 7 items												
Poor (0–2)	1			1			1			1		
Intermediate (3–4)	0.95	[0.82–1.10]	0.50	0.81	[0.70–0.94]	0.006	0.78	[0.67–0.91]	0.001	0.70	[0.58–0.83]	=0.001
Ideal (5–7)	1.01	[0.79–1.28]	0.92	0.71	[0.56–0.90]	0.001	0.70	[0.55–0.90]	0.004	0.56	[0.41–0.75]	<0.001
Behavioural component												
Poor (0–1)	1			1			1			1		
Intermediate (2)	0.81	[0.69–0.95]	0.01	0.71	[0.61–0.85]	<0.001	0.72	[0.61–0.85]	<0.001	0.66	[0.55–0.78]	<0.001
Ideal (3–4)	0.71	[0.59–0.86]	<0.001	0.55	[0.45–0.66]	<0.001	0.53	[0.44–0.65]	<0.001	0.40	[0.32–0.51]	<0.001
Biological component												
Poor (0–1)	1			1			1			1		
Intermediate (2)	1.16	[0.99–1.37]	0.06	0.98	[0.83–1.15]	0.79	0.97	[0.82–1.15]	0.78	1.11	[0.72–1.17]	0.61
Ideal (3)	1.10	[0.78–1.56]	0.56	0.97	[0.68–1.38]	0.87	1.00	[0.70–1.43]	0.96	1.02	[0.84–1.25]	0.77

Model 1 Adjusted for **Age** and **Gender**; Model 2 : Model 1 plus **education** and **living alone**; Model 3 : Model 2 plus **number** of health metrics at the intermediate level.

*High Level of Depressive Symptoms defined as Questionnaire of Depression 2nd version, Abridged ≥7 or being under antidepressant.

^†^p-value for polytomous logistic regression.

Odds ratio obtained by polytomous logistic regression.
