# Supplementary material for: High level of depressive symptoms as a barrier to reach an ideal cardiovascular health. The Paris Prospective Study III
Source: Sci Rep. 2016 Jan 8;6:18951. doi: 10.1038/srep18951 (PMC4705528; doi:10.1038/srep18951)
Supplement: Supplementary Information [file srep18951-s1.pdf]

High level of depressive symptoms as a barrier to reach an ideal cardiovascular health. The Paris Prospective Study III.

B. Gaye PhD (1,2), C Prugger MD PhD (1,2), MC Perier MSc (1,2), F Thomas PhD (3), M Plichart MD PhD (1,2,4), C Guibout PhD (1,2), C Lemogne MD PhD (2,5,6), B Pannier MD (3), P Boutouyrie MD PhD (1,2,7), X Jouven MD PhD (1,2,8), JP. Empana MD PhD (1,2)

(1) INSERM, UMR-S970, Paris Cardiovascular Research Center, Department of Epidemiology, Paris, France,

(2) Université Paris Descartes, Sorbonne Paris Cité, Faculté de Médecine, Paris, France

(3) Preventive and Clinical Investigation Center, Paris, France

(4) APHP, Hopital Broca, Department of Geriatry, Paris, France

(5) APHP, Georges Pompidou European Hospital, Psychiatry Department, Paris, France

(6) INSERM U894, Neuropsychiatry, Paris, France

(7) APHP, Georges Pompidou European Hospital, Pharmacology Departments, Paris, France

(8) APHP, Georges Pompidou European Hospital, Cardiology Department, Paris, France

SUPPLEMENTARY MATERIAL

Food Frequency questionnaire

Sugar sweetened-beverages were estimated through questions related to the daily amount of sweetened drinks (never, less than 0.5 liters, 0.5 to 1 liter per day). Daily sodium intake was estimated through questions related to the habitual composition of breakfast (none or only a drink, continental and English breakfast), the daily consumption of butter (never, once, two to four times and more than four times per day), dairy products (never, once, twice and more than twice a day), bread (never, less than 50g, 50 to 200g and more than 200g per day), meat (never, less than 100g, between 100 and 200g and more than 200g per day), cheese (never, one portion, two to three portions, and more than three portions per day), and weekly consumption (never, less than twice, two to four and more than four times a week) of eggs, cooked meats and starchy food, using the References manual of Food (ANSES : French Agency for Food, Environmental and Occupational Health & Safety, www. anses.fr). Information on the weekly consumption (never, less than twice, two to four and more than four times a week) of fish (all types), vegetables and fresh fruits were converted in average daily consumption.

Sensitivity analyses

Linear regression analysis

In exploratory descriptive statistics, a linear trend was observed between depressive symptoms score and CVH score in participants with a depressive symptoms score ≥7 only. We therefore run linear regression analysis in this subgroup and found that each additional point of the depressive symptoms score (from 7 to 13) was associated with a significantly lower *global* ideal cardiovascular health score (β =-0.05, p=0.004), *behavioural* score (β =-0.04, p=0.002) but not biological score (β =-0.009, p=0.32) after adjustment for age, sex, living alone and education. Furthermore, HLDS status was also inversely associated with the

global (β =-0.36, p<0.001) and the *behavioural* cardiovascular health score (β =-0.36, p<0.001) but not with the biological score (β <0.001, p=0.99) in linear regression analysis adjusted for age, sex, living alone and education.

Imputations of missing items of cardiovascular health

Missing data including depressive symptoms score, confounding variables (age, sex, education, living alone) and each health metric of the CVH were simultaneously imputed (15 times each) by multiple imputations with chained equations assuming that the data were missing at random. Predictive mean matching (continuous variable), logistic regression (binary variable), multinomial logistic regression (categorical variable) and ordinal logistic regression analyses (ordered categorical variable) were used. After imputation, the distribution of poor, intermediate and ideal cardiovascular health in the population remains virtually unchanged and was present in 41.39%, 48.88 % and 9.73% of the study participants respectively. Similarly, the decreased likelihood of having an ideal CVH in participants with HLDS remains unchanged (OR = 0.54; IC: [0.50-0.690] and OR = 0.78; IC: [0.74-0.84] for the *global* and *behavioural* CVH, respectively).

Stratified analysis by country of birth of the participants and of their parents

These analyses were conducted only for the *behavioural* component of the CVH. The prevalence of HLDS and ideal CVH, together with the association between HLDS and ideal CVH by country of birth of the participants (proxy for the migrant status) are reported on supplementary Table 2. Despite some heterogeneity in the distribution of both HLDS and ideal CVH across countries of birth, the association between HLDS and ideal CVH was fairly consistent across strata.

The prevalence of HLDS and ideal CVH, together with the association between HLDS and ideal CVH by country of birth of the parents of the participants (proxy for ethnicity) are reported on supplementary Table 3. Despite some heterogeneity in the distribution of both HLDS and ideal CVH across the ethnic groups, the association between HLDS and ideal CVH was fairly consistent across strata.

Cardiovascular health as a contributor to high level of depressive symptoms ?

When exploring the association between HLDS and CVH the other way around, we found that participants at the intermediate and ideal CVH level had respectively a 30% (OR=0.70; 95% CI: 0.58-0.84) and 43% (OR=0.57; 95% CI: 0.43-0.78) decreased likelihood of having HLDS after adjusting for age, gender, university education and living alone. This existed for the *behavioural* component of CVH only.

Exclusion of subjects with BMI<18 kg/m^2

After excluding the n=93 subjects with a BMI<18 kg/m^2, the results were virtually unchanged and after adjustment for age, sex, education level and living alone status, depressive symptoms were still inversely associated with the BMI metric (OR = 0.64; 95% CI: 0.52-0.80 after exclusion compared to OR = 0.63; 95% CI: 0.51-0.78 without exclusion of BMI<18 kg/m^2), behavioural CVH (OR = 0.54; 95% CI: 0.44-0.65 after exclusion compared to OR = 0.53; 95% CI: 0.44-0.65 without exclusion of BMI<18 kg/m^2) and global CVH (OR= 0.68; 95% CI: 0.52-0.88 after exclusion compared to OR = 0.70; 95% CI: 0.55-0.90 without exclusion of BMI<18 kg/m^2).

Supplementary Figure 1: Distribution of **Global** Cardiovascular Health by high levels of depressive symptoms status in the overall population (A) and by gender (B and C)

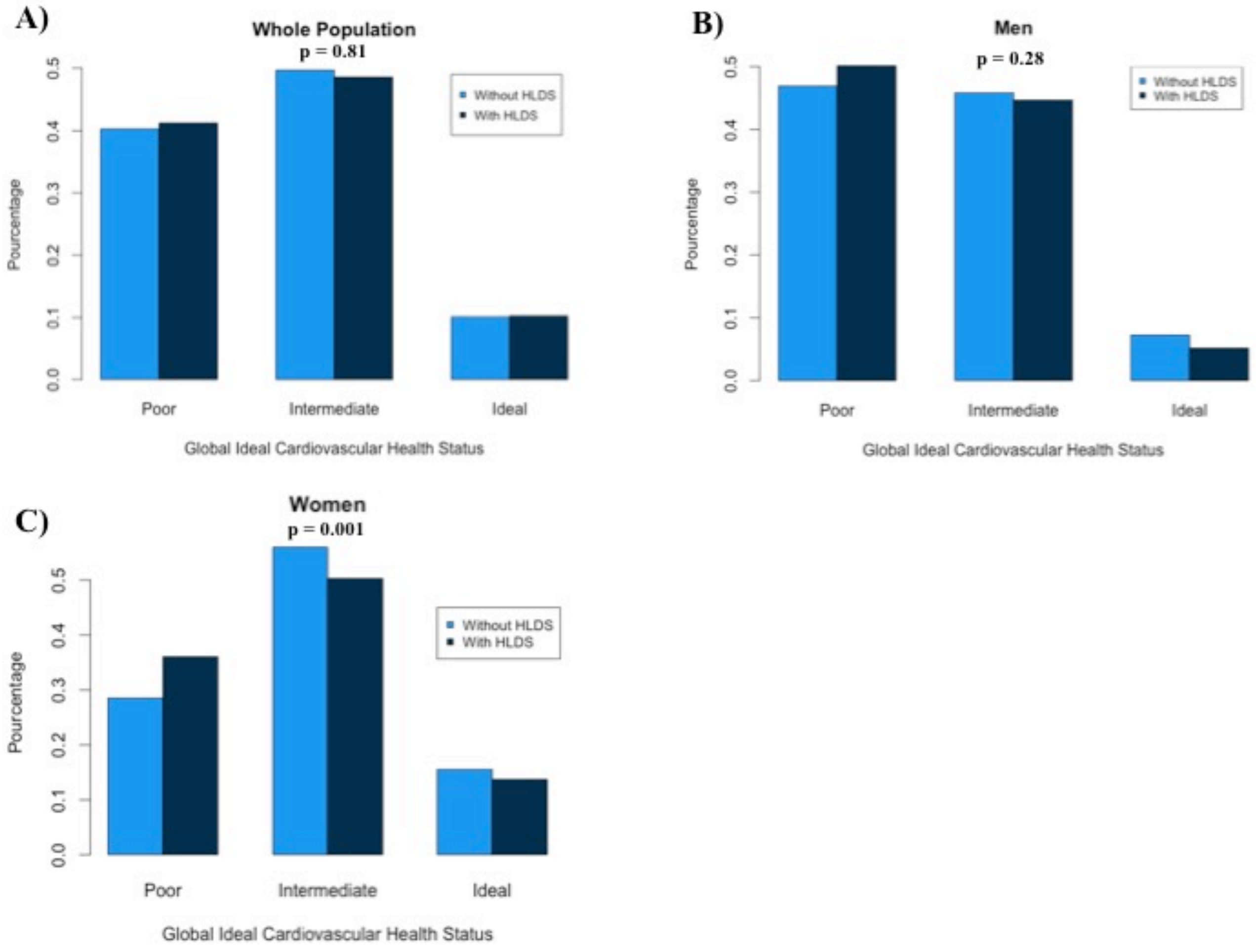

Supplementary Figure 2: Association between high level of depressive symptoms and **global** cardiovascular health: subgroups analysis.

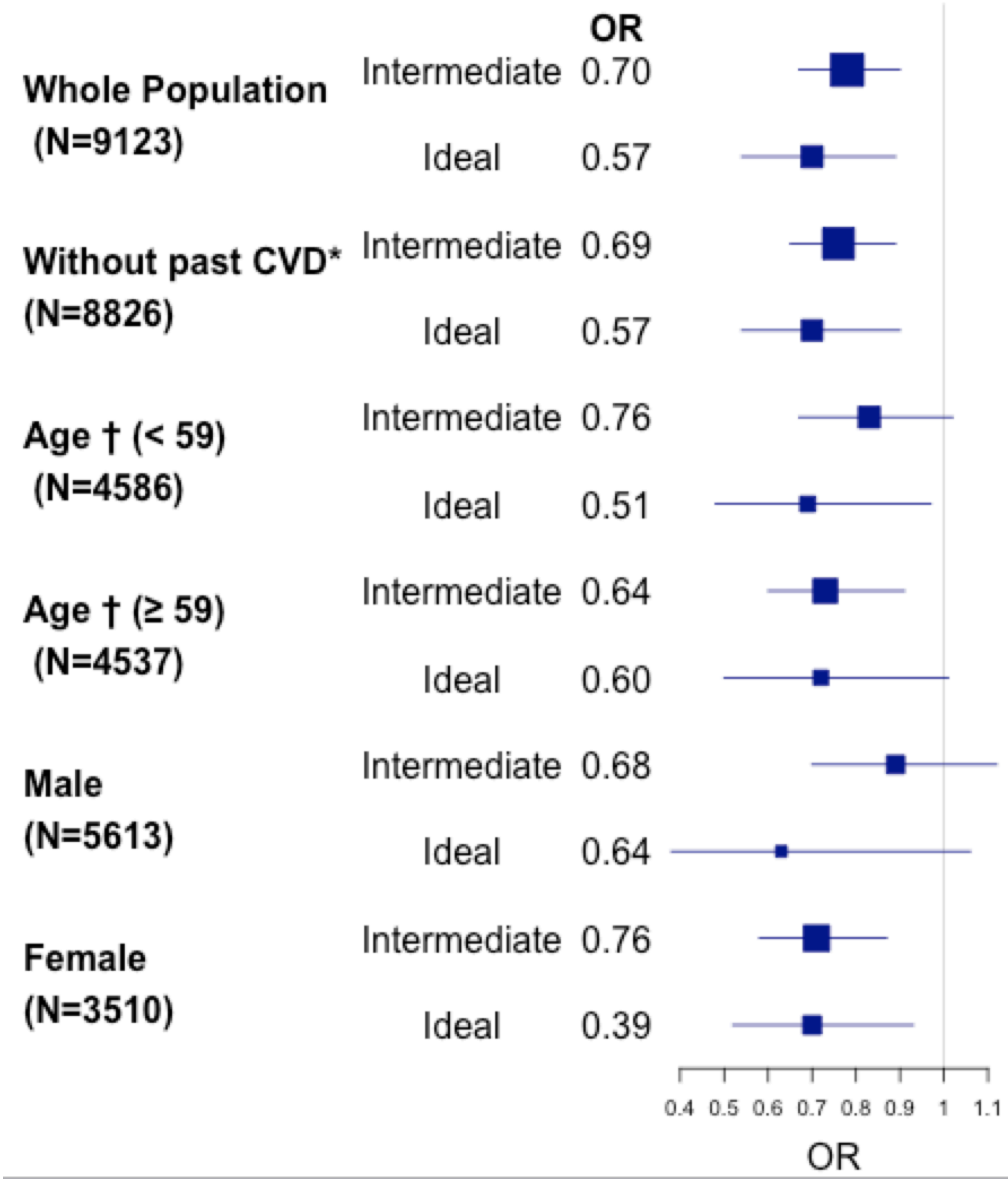

Supplementary Table 1

Variables	Excluded<br>N = 740	Not-Excluded<br>N = 9417	P*
Age	60.2 (+/-6.57)	59.6 (+/- 6.29)	*0.011*
Gender *Men*	442 (59.7%)	5759 (61.2%)	*0.467*
University education	419 (58.4%)	6459 (69.2%)	*0.001*
Self Rated Health>7	547 (75.7%)	7369 (78.3%)	*0.104*
Living Alone	233 (32.3%)	2445 (26.0%)	*0.001*
Smoker *Ideal*	583 (80.6%)	7639 (81.1%)	*0.774*
Body Mass Index kg/m^2	25.3 (+/-3.99)	25.2 (+/-3.71)	*0.488*
Physical Activity *Ideal*	376 (54.3%)	4572 (48.7%)	*0.005*
Fruits and Vegetables *Ideal*	45 (26.8%)	3372 (38.3%)	*0.003*
Fish *Ideal*	79 (43.6%)	3779 (42.6%)	*0.84*
Sugar *Ideal*	106 (58.6%)	5116 (58.0%)	*0.93*
Sodium *Ideal*	47 (36.4%)	3111 (63.6%)	*0.959*
Systolic blood pressure mmHg	132 (+/-16.3)	131 (+/-16.4)	0.176
Fasting Serum Glucose mg/dl	100 (+/-12.8)	101 (+/-14.4)	0.491
Total Cholesterol mg/dl	222 (+/-34.9)	221 (+/-36.4)	*0.6*
HLDS†	76 (10.3%)	898 (9.55%)	*0.526*
Personal history of CVD ‡	18 (2.53%)	241 (2.58%)	*1*
Personal history of Depression	95 (13.2%)	1211 (12.9%)	0.84
Treatment for blood pressure	120 (16.3%)	1299 (13.8%)	*0.069*
Lipid lowering treatment	98 (13.4%)	1205 (12.9%)	*0.703*
Treatment for depression	36 (4.92%)	498 (5.32%)	*0.7*

Data are reported as n (%) and mean (+/- standard deviation) for categorical and continuous variables respectively.

* Pearson chi-square test or Student test where appropriate

† High level of depressive symptoms defined as Questionnaire of Depression 2nd version, Abridged depressive symptoms score ≥ 7 or being under antidepressant.

‡ Cardiovascular disease

Supplementary Table 2 : Association between High Level of Depressive Symptoms and Behavioral Cardiovascular Health status by country of birth of the participants.

	**CVH status, N (%)**	**HLDS*, N (%)**	**OR† [95% CI]**	***p-value***	***p for interaction***
Country of birth of the participants					
**FRANCE**		713 (9.55)			
Poor	2882 (41.18)		1		
Intermediate	3423 (48.91)		0.70 [0.57-0.85]	<0.001	-
Ideal	693 (9.9)		0.56 [0.44-0.70]	<0.001	-
**EUROPE**		78 (12.3)			
Poor	227 (39.34)		1		
Intermediate	297 (54.47)		0.79 [0.41-1.52]	0.48	0.68
Ideal	53 (9.19)		0.67 [0.31-1.42]	0.29	0.72
**AFRICA**		96 (8.81)			
Poor	393 (39.74)		1		
Intermediate	498 (50.35)		0.80 [0.47-1.34]	0.40	0.73
Ideal	98 (9.91)		0.43 [0.21-0.87]	0.02	0.38
**ASIA**		20 (7.55)			
Poor	80 (33.61)		1		
Intermediate	125 (52.52)		0.67 [0.18-2.47]	0.55	0.97
Ideal	33 (13.87)		0.54 [0.13-2.14]	0.38	0.97
**AMERICA**		16 (12.5)			
Poor	34 (28.33)		1		
Intermediate	62 (51.67)		0.29 [0.06-1.45]	0.13	0.36
Ideal	24 (20)		0.43 [0.10-1.76]	0.24	0.93
**FRENCH OVERSEAS**		14 (6.17)			
Poor	81 (40.91)		1		
Intermediate	105 (53.03)		1.30 [0.34-4.90]	0.68	0.37
Ideal	12 (6.06)		0.27 [0.03-2.65]	0.26	0.51

The country of birth of the participants was missing for n= 288 individuals and 9 subjects only were born in Oceania, so that analysis was carried out in n=9120 participants

* High level of depressive symptoms defined as Questionnaire of Depression 2nd version, Abridged depressive symptoms score ≥ 7 or being under antidepressant.

† Odds ratio and 95% Confident Interval were obtained by polytomous logistic regression and were adjusted for age and gender education and living alone status.

Supplementary table 3. Association between High Level of Depressive Symptoms and Behavioral Cardiovascular Health status by country of birth of the parents' participants.

	CVH status, N(%)	HLDS*, N(%)	OR† [95% CI]	*p-value*	*p for interaction*
Country of birth of the parents' participants					
**FRANCE**		595 (9.33)			
Poor	2971 (49.67)		1		
Intermediate	2421 (40.48)		0.63 [0.51-0.78]	<0.001	-
Ideal	589 (9.85)		0.53 [0.41-0.68]	<0.001	-
**EUROPE**		93 (13.36)			
Poor	267 (41.52)		1		
Intermediate	322 (50.08)		0.79 [0.44-1.43]	0.45	0.46
Ideal	54 (8.4)		0.66 [0.33-1.30]	0.23	0.47
**AFRICA**		88 (10.14)			
Poor	316 (40.56)		1		
Intermediate	388 (49.81)		0.87 [0.50-1.50]	0.62	0.32
Ideal	75 (9.63)		0.45 [0.21-0.94]	0.03	0.61
**ASIA**		19 (7.76)			
Poor	72 (32.73)		1		
Intermediate	113 (51.36)		0.76 [0.21-2.74]	0.68	0.79
Ideal	35 (15.91)		0.46 [0.11-1.90]	0.28	0.95
**FRENCH OVERSEAS**		15 (6.7)			
Poor	82 (41.62)		1		
Intermediate	103 (52.28)		1.70 [0.46-6.23]	0.41	0.22
Ideal	12 (6.09)		0.31 [0.03-3.10]	0.32	0.54
**FRANCE-MIXED‡**		68 (8.99)			
Poor	285 (40.37)		1		
Intermediate	345 (48.87)		0.91 [0.49-1.71]	0.68	0.20
Ideal	76 (10.76)		0.70 [0.33-1.48]	0.36	0.44
**Others**		19 (10.73)			
Poor	62 (37.58)		1		
Intermediate	75 (45.45)		0.57 [0.14-2.24]	0.42	0.80
Ideal	28 (16.97)		0.80 [0.21-3.02]	0.75	0.46

* High level of depressive symptoms defined as Questionnaire of Depression 2nd version, Abridged depressive symptoms score ≥ 7 or being under antidepressant.

† Odds ratio and 95% Confident Interval were obtained by polytomous logistic regression and were adjusted for age and gender education and living alone status.

‡ One parent's country of birth is France. The country of birth of the parents' participants was missing for n= 726 individuals
